# Supplementary material for: Association of Selenoprotein and Selenium Pathway Genotypes with Risk of Colorectal Cancer and Interaction with Selenium Status
Source: Nutrients. 2019 Apr 25;11(4):935. doi: 10.3390/nu11040935 (PMC6520820; doi:10.3390/nu11040935)

ASSOCIATION OF SELENOPROTEIN AND SELENIUM PATHWAY GENOTYPES WITH RISK OF COLORECTAL CANCER AND INTERACTION WITH SELENIUM STATUS

AUTHORS

VERONIKA FEDIRKO[1], MAZDA JENAB[2], CATHERINE MÉPLAN[3], JEB S. JONES[1], WANZHE ZHU[1], LUTZ SCHOMBURG[4], AFSHAN SIDDIQ[5], SANDRA HYBSIER[4], KIM OVERVAD[6], ANNE TJØNNELAND[7], HANANE OMICHESSAN[8,9], VITTORIO PERDUCA[8,9,10], MARIE-CHRISTINE BOUTRON-RUAULT[8,9], TILMAN KÜHN[11], VERENA KATZKE[11], KRASIMIRA ALEKSANDROVA[12], ANTONIA TRICHOPOULOU[13], ANNA KARAKATSANI[13,14], ANASTASIA KOTANIDOU[13,15], ROSARIO TUMINO[16], SALVATORE PANICO[17], GIOVANNA MASALA[18], CLAUDIA AGNOLI[19], ALESSIO NACCARATI[20], BAS BUENO-DE-MESQUITA[21,22,23,24], ROEL C.H. VERMEULEN[25], ELISABETE WEIDERPASS[26,27,28,29], GURI SKEIE[29], THERESE HAUGDAHL NØST[29], LEILA LUJAN-BARROSO[30], J. RAMÓN QUIRÓS[31], JOSÉ MARÍA HUERTA[32,33], MIGUEL RODRÍGUEZ-BARRANCO[33,34], AURELIO BARRICARTE[33,35,36], BJÖRN GYLLING[37], SOPHIA HARLID[38], KATHRYN E. BRADBURY[39], NICK WAREHAM[40], KAY-TEE KHAW[41], MARC GUNTER[2], NEIL MURPHY[2], HEINZ FREISLING[2], KOSTAS TSILIDIS[5,42], DAGFINN AUNE[5], ELIO RIBOLI[5], JOHN E. HESKETH[3], DAVID J. HUGHES[43]

Supplementary Figure S1: Selenium pathway genetic analysis flowchart

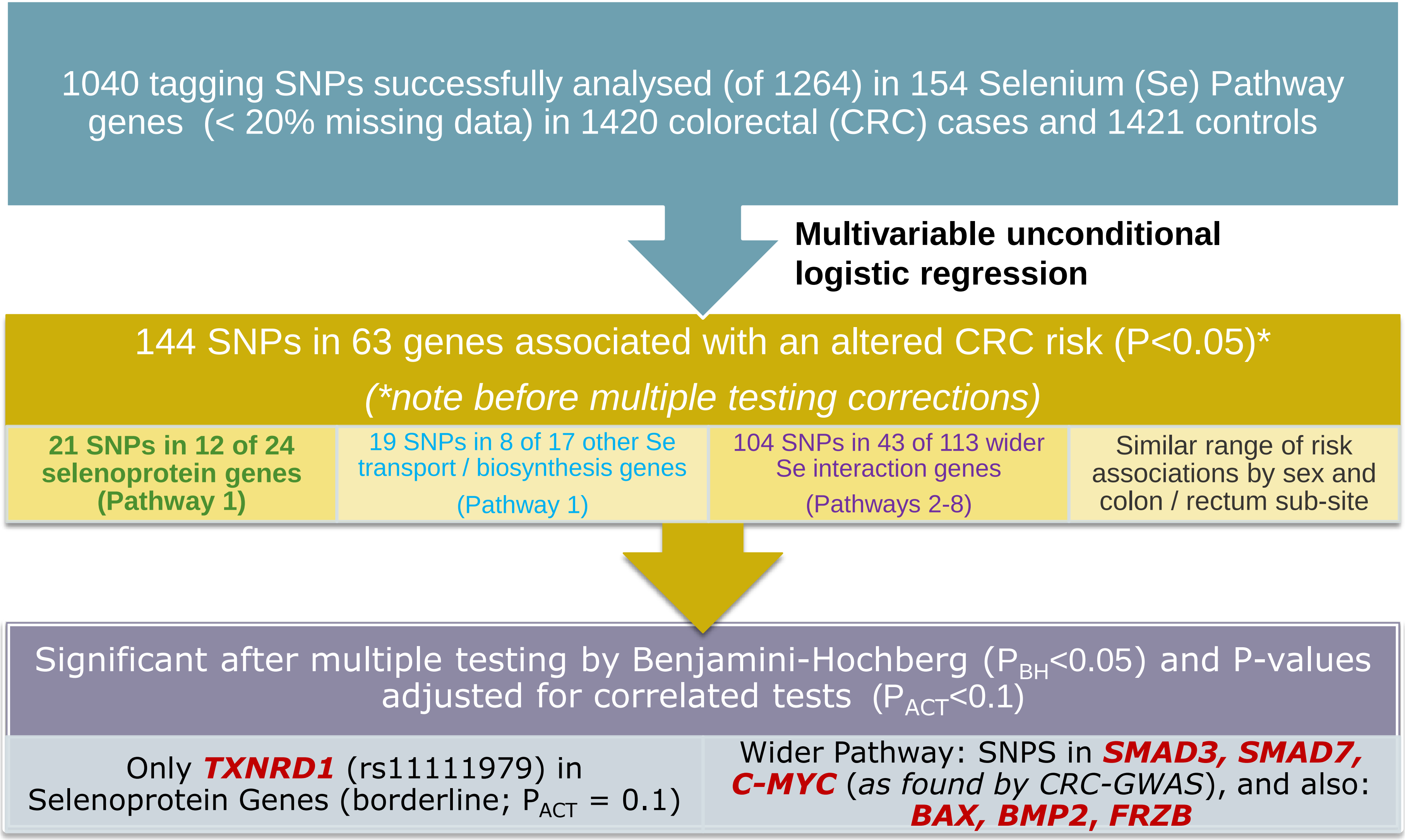

Supplement: Supplementary file 1 [file nutrients-11-00935-s001.zip › Supp Figure S1 Selenium pathway genetic analysis summary.pdf]
